# Paper-Based Point-of-Care Testing of SARS-CoV-2

**DOI:** 10.3389/fbioe.2021.773304

**Published:** 2021-11-29

**Authors:** Yuan Jia, Hao Sun, Jinpeng Tian, Qiuming Song, Wenwei Zhang

**Affiliations:** ^1^ College of New Materials and New Energies, Shenzhen Technology University, Shenzhen, China; ^2^ School of Mechanical Engineering and Automation, Fuzhou University, Fuzhou, China; ^3^ Sino-German College of Intelligent Manufacturing, Shenzhen Technology University, Shenzhen, China

**Keywords:** paper-based, point-of-care, COVID-19 diagnostics, immunoassay, CRISPR

## Abstract

The COVID-19 pandemic has resulted in significant global social and economic disruption. The highly transmissive nature of the disease makes rapid and reliable detection critically important. Point-of-care (POC) tests involve performing diagnostic tests outside of a laboratory that produce a rapid and reliable result. It therefore allows the diagnostics of diseases at or near the patient site. Paper-based POC tests have been gaining interest in recent years as they allow rapid, low-cost detection without the need for external instruments. In this review, we focus on the development of paper-based POC devices for the detection of SARS-CoV-2. The review first introduces the principles of detection methods that are available to paper-based devices. It then summarizes the state-of-the-art paper devices and their analytical performances. The advantages and drawbacks among methods are also discussed. Finally, limitations of the existing devices are discussed, and prospects are given with the hope to identify research opportunities and directions in the field. We hope this review will be helpful for researchers to develop a clinically useful and economically efficient paper-based platform that can be used for rapid, accurate on-site diagnosis to aid in identifying acute infections and eventually contain the COVID-19 pandemic.

## Introduction

The new coronavirus (SARS-CoV-2) caused pneumonia (COVID-19) outbreak has quickly spread the world and developed into a global pandemic. It has turned the lives of billions of people upside down and created chaos in healthcare, economic, and social domains (Mofijur et al., 2021). Looking back on history, although other infectious disease outbreaks have caused serious social and economic repercussions, such as the Middle East respiratory syndrome (MERS-CoV) and severe acute respiratory syndrome (SARS-CoV), none of them have posed the same level of threat to mankind as COVID-19 (Sohrabi et al., 2020; Wang et al., 2021).

**FIGURE 1:**
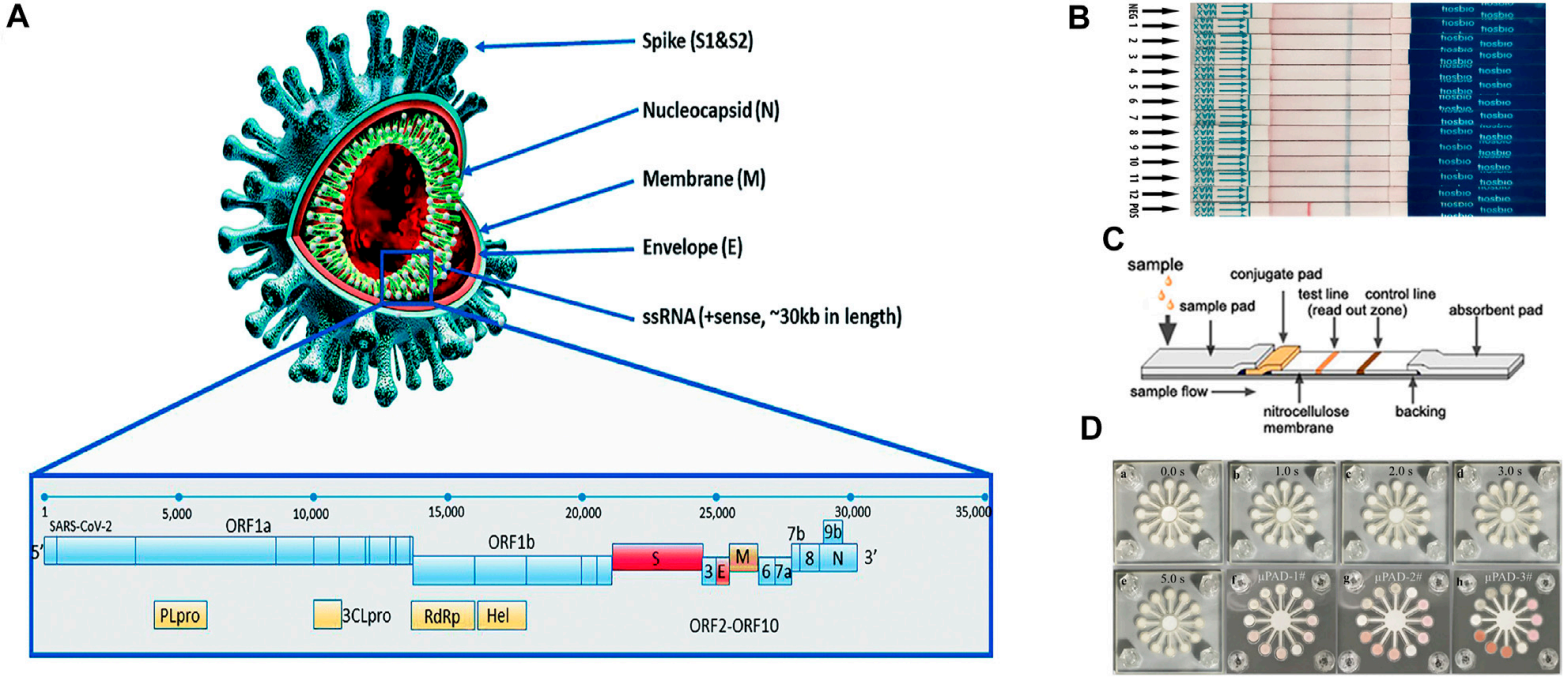
**(A)** Structural proteins of SARS-CoV-2: envelope (E), membrane (M), spike (S), helicase (Hel), and nucleocapsid (N). Adapted with permission from (Kubina and Dziedzic, 2020) under Creative Commons license; **(B)** dipstick paper test. Adapted with permission from (Zheng Y.-Z. et al., 2021) under Creative Commons license; **(C)** lateral flow paper test. Adapted with permission from (He et al., 2016) under Creative Commons license; **(D)** microfluidic paper-based analytical devices. Adapted with permission from (Sun et al., 2019) under Creative Commons license.

SARS-CoV-2 is a positive-stranded non-segmented single-stranded RNA virus with a 29.9 kb genome length (Mousavizadeh and Ghasemi, 2021) (Figure 1A). Despite being a variant of a large group of viruses that cause the common cold, MERS-CoV, and SARS-CoV (Zhou et al., 2020), SARS-CoV-2 has four distinctively interconnected traits: high reproduction number, a large number of asymptomatic or mild symptom cases, relatively long incubation period, and survival of the virus in some environments (World Health Organization, 2020). In addition, it has been found that asymptomatic patients may have the same viral load as the symptomatic patients, thus making them both susceptible as a source of viral transmission, even in the early stages of the infection (Gao et al., 2021). Vaccines have been developed to prevent infection. However, SARS-CoV-2 mutations in the spreading of the disease pose a great challenge to the efficacy of vaccines (Dos Santos, 2021). Therefore, at the current stages of the pandemic, the development of diagnostic technologies is of critical importance as fast and accurate identification of early case clusters is still the key intervention measure to stop transmission. Nevertheless, because 51–67% of the world’s population today lack equitable access to essential public health services (United Nations, 2020), the economically efficient and easy-to-use paper-based POC tests have been given serious consideration as a potential method for COVID-19 diagnostics.

Paper was first considered as a viable substrate material for enabling fluidic control for chromatography (Muller and Clegg, 1949). The first fully operable paper-based POC device was introduced by Whiteside’s group in 2007 (Martinez et al., 2007; Sachdeva et al., 2021b). Since then, thanks to advantages including self-driven flow, a relatively high surface to volume ratio, ubiquity, and superb biocompatibility (Gong and Sinton, 2017), different types of paper devices such as dipstick tests (Yetisen et al., 2013), lateral flow devices (Huang et al., 2016; Zhou et al., 2019; Wu et al., 2020), and microfluidic paper-based analytical devices (µPADs) (Morbioli et al., 2017; Komatsu et al., 2018) have been extensively studied in academia and some of them have been successfully converted to commercial products (Figures 1B–D). They represent one of the promising technologies featuring cost-effectiveness, portability, and accessibility that are particularly useful in the field of POC medical diagnostics (Jia et al., 2018), environmental testing (Sun et al., 2019), and food quality assessment (Li et al., 2012). In the diagnostics of infectious diseases, paper-based POC devices have been demonstrated for the detections of human norovirus (Han et al., 2016), human papillomavirus (HPV) (Rodriguez et al., 2016), Hepatitis B (Li et al., 2015), West Nile virus (Channon et al., 2018), *etc*. Additionally, paper-based POC devices have shown comparable performances to conventional instruments in terms of both sensitivity and accuracy, while providing a faster turnaround time (Gong and Sinton, 2017). Therefore, this technology can be leveraged for detecting SARS-CoV-2 detection.

Given the urgency of the subject matter, several reviews on COVID-19 POC diagnostics methods have been published, which have provided valuable insights into the developmental progress and the outstanding issues for commercialization (Zhu H. et al., 2020; Choi, 2020; Wang et al., 2021). However, almost all of the reviews focused on the POC aspect, which applies to both paper-based platforms and conventional microfluidic platforms such as PDMS, PMMA, and others. This on one hand extends the scope of the existing reviews, but on the other hand, can only provide limited information on paper-based methods. Additionally, the few available reviews dedicated to paper-based methods are written in the early stage of the pandemic, and great progress has been made in paper-based POC devices since then (Antiochia, 2021). Therefore, a comprehensive review of paper-based COVID-19 diagnostics methods is still needed. In this review, we focus on the development of paper-based POC devices for the detection of SARS-CoV-2. The review first introduces the available paper-based SARS-CoV-2 detection methods that include immunoassay tests, nucleic acid amplification tests (NAAT), and Clustered Regularly Interspaced Short Palindromic Repeats/CRISPR associated proteins (CRISPR/Cas) systems. It then presents the state-of-the-art paper devices and their analytical performances, as well as the advantages and drawbacks. Finally, limitations of the paper devices are discussed, and prospects are given with the hope to identify research opportunities and directions in the field.

## Paper-Based SARS-CoV-2 Diagnostic Methods

**FIGURE 2:**
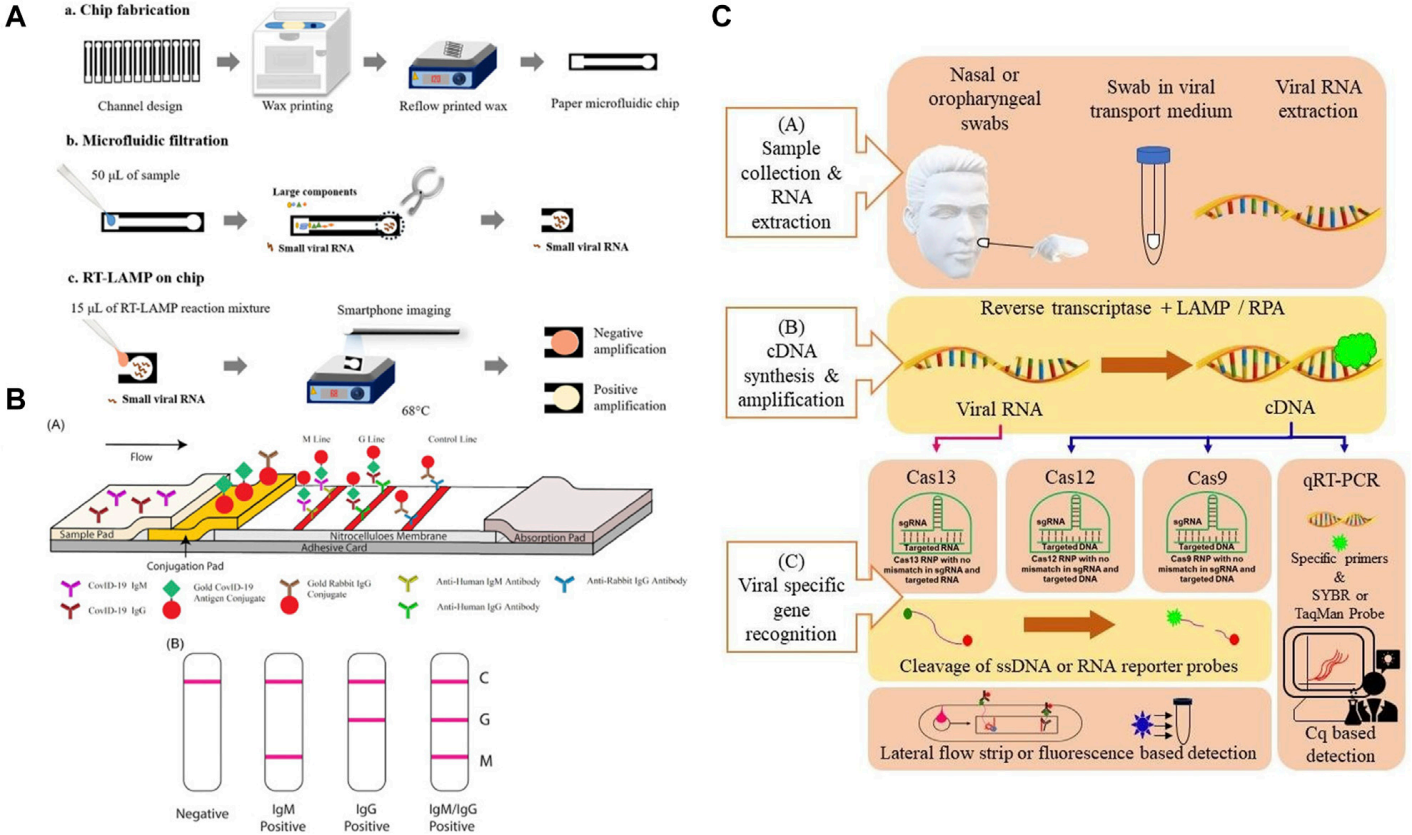
**(A)** Principle of a typical Nucleic acid amplification test. Adapted with permission from (Kaarj et al., 2018) under Creative Commons license; **(B)** principle of a typical lateral flow test. Adapted with permission from (Li et al., 2020) under Creative Commons license; **(C)** principle of a CRISPR test. Adapted with permission from (Javalkote et al., 2020) under Creative Commons license.

According to the World Health Organization (WHO), because of its relatively high sensitivity and specificity, reverse transcription-polymerase chain reaction (RT-PCR)-based SARS-CoV-2 RNA detection from respiratory samples (e.g., nasopharynx) is the standard method for diagnosis. However, the conventional method still has the disadvantages of requiring expensive laboratory instruments and skilled laboratory personnel, which can be difficult to obtain in an underdeveloped area (Habibzadeh et al., 2021). Therefore, paper-based POC diagnostic methods have the potential to complement the conventional diagnostic method. The current paper-based diagnostic methods can be grouped based on the detection targets, including (a) detection of SARS-CoV-2 viral genes, (b) detection of viral antigens (c) detection of antibodies (serological test), each of them serves a different diagnostic need. The viral gene and antigen detection tests detect present viral infections, whereas serological tests determine prior infections (Antiochia, 2021). Of these, the immunoassay tests in particular lateral flow immunoassay tests (LFIA) are designed to target antibodies and antigens. The other methods including NAAT and CRISPR/Cas detect viral genes but rely on different detection approaches. In this section, we introduce the available paper-based SARS-CoV-2 diagnostic methods (Figure 2).

### Lateral Flow Immunoassay

Relying on the natural wicking property of paper, LFIA is the most commercially successful paper-based POC diagnostic device to date. Although the most commonly known LFIA is the home pregnancy test, it has developed into the go-to method for the rapid detection of various biomarkers and substances (Liu and Rusling, 2021). The underlying principle of the LFIA is to utilize the specific binding between antibodies and antigens, proteins, or hormones. Unlike conventional PCR, LFIA is amplification-free, therefore much easier to operate. The typical device design of an LFIA consists of a sample pad, a conjugation pad, and an absorbent pad. Target analyte solution is first absorbed in the sample pad and moved toward the conjugation pad through cellulose membrane by capillary force. Here, a coloring agent labeled antibody or DNA/RNA oligonucleotide that is specific to the analyte forms a conjugate with the analyte and is transported along the membrane. Affinity ligands that are specific to the target analyte/conjugated antibody complex are immobilized in designated zones, which are called “test lines”. When the solution containing the complex reaches the test line, a signal in terms of a color change is generated as soon as the analyte is captured by the bioreceptor. The solution continues to flow across the membrane until it reaches the “control line”. The control line contains affinity ligands that will capture the conjugate with or without the presence of an analyte in solution to confirm that the assay is working properly. Finally, the excess solution reaches the absorbent pad at the end of the strip and thus completing the assay (Yuce et al., 2021). For the case of SARS-CoV-2, its characteristic protein such as N protein and S protein can specifically bind to commonly used receptors IgG or IgM (Chen et al., 2020).

### Nucleic Acid Amplification Tests

LFIAs primarily use antibodies as recognition elements and focus on detecting other antibodies, proteins, and hormones, etc (Koczula and Gallotta, 2016). However, because there is no possibility to enhance the response of LFIAs by enzyme reactions, the sensitivity of LFIAs is often limited (Mahmoudi et al., 2019). Additionally, nucleic acids are less prone to integrity loss. Therefore, nucleic acid detection is a more accurate approach when compared with immunoassays (Reid et al., 2020). The working principle of NAATs is to first generate copies of the target gene sequence through amplification and subsequently use a detection probe to produce a signal. The amount of signal produced is directly proportional to the nucleic acid concentration (Ballard and Ozcan, 2018). Therefore, similar to the conventional PCR-based testing, NAATs can be used to specifically identity target gene in a relatively low concentration and are regarded as one of the most sensitive of all available paper-based POC tests for infectious disease diagnosis (Kaur and Toley, 2018). Also similar to conventional PCR testing, there are three main steps in a NAAT assay: sample preparation (including extraction purification), amplification, and detection. Of these, the sample preparation step often involves the lysis of biological samples to release nucleic acids, which are then purified by separating from other lysed components and eluting (Magro et al., 2017). Nasopharyngeal swab or throat swab of sputum is frequently used for upper respiratory tract specimen collection, and subsequent centrifugation and filtration procedures may be required (Gao et al., 2021). During the amplification step, the nucleic acids are replicated from a template to obtain a large number of specific nucleic acid fragments for subsequent detection. Amplification in NAATs can be done either by on-chip PCR or isothermal amplification. Similar to the conventional method, the on-chip PCR requires thermocycling to mediate DNA melting, followed by primer annealing and extension (Niemz et al., 2011). Contrary to PCR, isothermal amplification is developed to replace the thermal cycling steps to simplify and lower the cost of NAATs. It copies target nucleic acids by using enzymes to denature the double strands of DNA and specially designed primer sets to amplify a target sequence (Gill and Ghaemi, 2008). Isothermal amplification techniques including loop-mediated isothermal amplification (LAMP), nucleic acid sequence-based amplification (NASBA), transcription-mediated amplification (TMA), rolling circle amplification (RCA), and recombinase polymerase amplification (RPA) have been used for developing COVID-19 diagnostic tests (Hosseini et al., 2020). the detection of NAATs is accomplished either by tagging the amplified nucleic acids with specific reporter molecules such as fluorescence-based dyes or measuring turbidity, which is a by-product (magnesium pyrophosphate) of DNA polymerization (Lee et al., 2008).

Combining the lateral flow technique and target gene amplification, NALF is the most commonly found paper-based NAAT device type. Currently, there are two primary methods for performing lateral flow nucleic acid tests, NALF and nucleic acid lateral flow immunoassay (NALFIA). Both NALF and NALFIA methods combine the power of enzymatic exponential amplification of the target gene sequence with the sensitivity and ease of use offered by the LFIA technique (Jauset-Rubio et al., 2016a). Although these methods detect target nucleic acids via hybridization to complementary sequences, their binding principles are different as NALF directly detects DNA exploiting capture and labeled reporter oligonucleotide probes, whereas NALFIA detects hapten-labeled DNA using capture and labeled reporter antibodies or streptavidin (Jauset-Rubio et al., 2016b). A typical workflow of lateral flow nucleic acid tests comprises nucleic acid extraction from biological samples, nucleic acid amplification, and the detection of an analyte through LFAs using labeled color-changing agents (Zheng C. et al., 2021).

### Clustered Regularly Interspaced Short Palindromic

The CRISPR system is originally discovered in bacteria as a defense mechanism to fight against foreign nucleic acid invasions. When the guide RNA (gRNA) recognizes the genetic sequence of the foreign nucleic acids, it activates the Cas endonuclease to cut off the intruder’s genetic sequence (Wright et al., 2016). CRISPR so far has discovered three major systems including Cas9, Cas13, and Cas12 (Li et al., 2019a). However, it is only discovered recently that it can be considered as a novel diagnostic tool for the detection of nucleic acids. For instance, Cas12a is one of the nucleases that possess collateral cleavage activities on single-stranded DNA (ssDNA) under the guidance of a gRNA. After recognizing its specific DNA targets, the Cas12a can be activated, cleave the collateral ssDNA reporter and generate a fluorescent signal (Gootenberg et al., 2018). It offers high sensitivity, specificity, simplicity and has been successfully applied to pathogen detection, cancer mutation detection, etc (Gootenberg et al., 2017). Recently, CRISPR has been combined with paper-based lateral flow techniques and used as a diagnostic tool for rapid detection of COVID-19. To obtain a sufficient amount of the target gene, an amplification step is usually included. To avoid the use of external a thermal cycler, isothermal amplification such as LAMP and RPA are also commonly used with the CRISPR/Cas systems.

## Application of Paper-Based POC Tests for COVID-19

In this section, state-of-the-art paper-based POC devices are grouped by their detection methods and reviewed. Since both NAATs and lateral flow nucleic acid tests differ only in paper device design, they are categorized in the same group. The advantages and disadvantages of the tests are individually discussed. In addition, tables are listed in the end to give a quick comparison between each work in each group.

### Paper-Based Immunoassay Tests

According to the classification of detection targets, immunoassay tests are mainly used for antigen and antibody detection. Because of their advantages in operation simplicity, cost-effectiveness, and rapid detection time, paper-based immunoassay tests are usually used to complement SARS-CoV-2 nucleic acid detection. Based on different detection techniques, immunoassays mainly can be divided into LFIA, enzyme-linked immunosorbent assay (ELISA), and chemiluminescence.

Utilizing LFIA, Li and co-works developed a paper-based POC device for the detection of SARS-CoV-2 antibodies in human blood samples with a visual readout. SARS-CoV-2 Spike Glycoprotein (S1) recombinant antigen, which was conjugated to colloidal gold nanoparticles (AuNP), was chosen to bind to both SARS-CoV-2 immunoglobulin M (IgM) and immunoglobulin G (IgG) antibodies. When patient blood samples were introduced to the paper strip, anti-SARS-CoV-2 IgG and IgM antibodies, if present in the specimen, would specifically bind to the antigen on the conjugate pad, and the complex would subsequently bind to the antibodies on the test line. The detection sensitivity and specificity were obtained using whole blood samples collected from 397 PCR confirmed COVID-19 patients and 128 healthy individuals. The overall testing sensitivity was 88.66% and specificity was 90.63%, and the total test time was 15 min (Li et al., 2020). Using a direct antigen-antibody interactions principle, Wen and co-workers developed a LFIA by fixing SARS-CoV-2 nucleocapsid protein (N) to the surface of the strip and using anti-human IgG functionalized AuNPs to detect antibodies in serum. The IgG antibody will specifically bind to the anti-human IgG mAbs presented in the conjugation pad. The resulting complex subsequently binds to the N protein to induce a color change at the test line. The clinical test revealed a 69.1% sensitivity and a 100% specificity using samples collected from 55 clinically confirmed patients and 30 normal human sera (Wen et al., 2020). Despite their usefulness, reports have indicated that AuNP-LFIAs typically are limited by their relatively low sensitivity and high false-negative rates. To address these, Huang and co-workers carried out optimizations of the AuNP-LFIA include altering the pH value and the amount of antihuman IgM, the assay achieved higher sensitivity and specificity of 100 and 93.3%, respectively. However, the sample size in this work is relatively small, which consists of human serum samples collected from five confirmed patients and 14 healthy individuals (Huang et al., 2020). Zhou and co-workers first proposed the use of a polymer-type structure-directing agent polyethyleneimine to assist in copper *in-situ* growth (PEI-CISG) on the surface of AuNP probes. Because PEI-CISG can control the shape and size of the resultant Au-CuNP core-shell nanostructures, the detection signal of the conventional AuNP-LFIA can be amplified. The authors incorporated the PEI-CISG into a commercial AuNP-LFIA SARS-CoV-2 detection kit, and the PEI-CISG treated LIFA successfully detected all previous false-negative samples (Zhou et al., 2021b). In a later work, the same group developed an alternative fluorescent nanomaterial to replace the conventional AuNPs, the quantum dot nanobeads (QBs). Because of their high luminescent intensity and resistance to matrix interference, the QB-LFIA detected SARS-CoV-2 total antibodies in 69 human serum samples with a sensitivity of 97.1% and a specificity of 100% (Zhou et al., 2021a). In another study, lanthanide-doped polystyrene nanoparticles (LNPs) were used as a fluorescent reporter in a LFIA strip to detect anti-SARV-CoV-2 IgG in human serum. A recombinant N-phosphoprotein was used to specifically capture the target IgG. The test showed a 100% sensitivity and an 88% specificity using samples collected from seven confirmed patients and 51 normal human serum (Chen et al., 2020).

Instead of nitrocellulose based-LFA strips, Kim and co-workers developed a vertical flow paper device to detect SARS-CoV-2 antibodies. In this work, SARS-CoV-2 N protein was fused with a cellulose-binding domain (CBD) for the detection of target antibodies as cellulose-CBD interaction would allow rapid immobilization of SARS-CoV-2 antigens on cellulose paper strips. Also, leveraging the multivalency of target antibodies (both IgG and IgM), a double-antigen sandwich format was developed and the functionalized antigens were used as both capture and reporter reagents, replacing the anti-human antibodies. Specifically, the capture and reporter reagents are NP-CBD and biotinylated NP (NP-biotin), respectively. Biotin was used to conjugate NP with horseradish peroxidase (HRP) via a biotin−streptavidin (SA) interaction. Thus, HRP could be associated with CBD to form enzyme-antibody-CBD sandwich complexes as the presence of SARS-CoV-2 antibodies link the capture and reporter reagents. Samples were collected from three confirmed patients’ serum and a 96.2 and 93.9% sensitivity was reported for IgG and IgM detection, respectively (Kim et al., 2021). Finally, instead of lateral flow strips, Kasetsirikul and co-workers developed a paper-based ELISA test for the detection of SARS-CoV-2 humanized antibodies. Circular paper reactions wells were fabricated by laser cutting and were then laminated for easy sample handling. Recombinant SARS-CoV-2 nucleocapsid antigen was coated on the reaction wells to capture the SARS-CoV-2 humanized antibody (Kasetsirikul et al., 2020).

Besides SARS-CoV-2 antibodies, paper-based devices for antigen detection have also been developed. Compared with antibodies, which usually do not appear in the early stage of infection, viral antigen has potential to become a viable target for early diagnosis of SARS-CoV-2 infection. Reports have indicated that the NP antigen is one of the best early diagnostic markers in SARS-CoV and can be detected up to 1 day before the appearance of clinical symptoms (Che et al., 2004). Based on this, Diao and coworkers proposed a lateral flow device based on fluorescence immunochromatographic principle to detect the SARS-CoV-2 NP antigen. Fluorescent microparticles-labeled mouse anti-NP antibody was immobilized on a nitrocellulose membrane for detecting NP antigen in nasopharyngeal swabs and urine samples within 10 min. By comparing with RT-PCR testing, a sensitivity of 68% and a specificity of 100% were achieved out of 251 patients (Diao et al., 2021). In another work, a dipstick test has been developed by Grant and co-workers for SARS-CoV-2 NP antigen detection. The dipstick test consists of a nitrocellulose membrane and a wicking pad, without sample and conjugate pads. Optical detection was achieved by conjugating red latex particles to commercially available polyclonal antibodies. However, no clinical samples were used in this study due to the specificity concern of the antibodies (Grant et al., 2020). Based on the chemiluminescence principle, Liu and co-workers developed a paper-based test for the detection of SARS-CoV-2 spike antigen (S antigen). Instead of using natural proteases such as HRP commonly used in paper-based enzymatic chemiluminescence assays, the authors developed a Co–Fe@hemin-peroxidase nanozyme that can amplify immune reaction signals. Compared with HRP, the nanozyme is more stable and much suitable for POC testing. The assay achieved an ELISA comparable S antigen detection limit while reducing the operation time to approximately 15 min. However, no clinical samples were used in this study (Liu et al., 2021a). Hristov and co-workers developed a paper-based multiplexed antigen test that can differentiate S proteins from different coronaviruses including SARS-COV-1, SARS-COV-2, and CoV-HKU1 as well as spike protein variants from SARS-CoV-2. The test relied on a sandwich immunoassay and antibody cross-reactivity for antigen-specific test patterns. In their work, six antibodies were evaluated for antigen binding and sandwich immunoassay formation. By identifying specific binding patterns that are achieved by designating antibody-antigen reaction locations, fractions of the same spike protein can then be differentiated (Hristov et al., 2021). Finally, Yakoh and co-workers developed a paper-based electrochemical test for the detection of either SARS-CoV-2 anybody or antigen. Unlike previous LFIA tests, the electrochemical system is label-free, therefore does not require the use of reporter-labeled antibodies. Instead, the SARS-CoV-2 spike protein-containing receptor-binding domain is immobilized to capture incoming SARS-CoV-2 antibodies. The sensing scheme relies on the disruption of the redox conversion ([Fe (CN)6] 3-/4-) triggered by immunocomplex formation between the spike protein and target antibody. The test can also be extended for direct detection of the spike protein antigen of SARS-CoV-2 (Yakoh et al., 2021).

**FIGURE 3:**
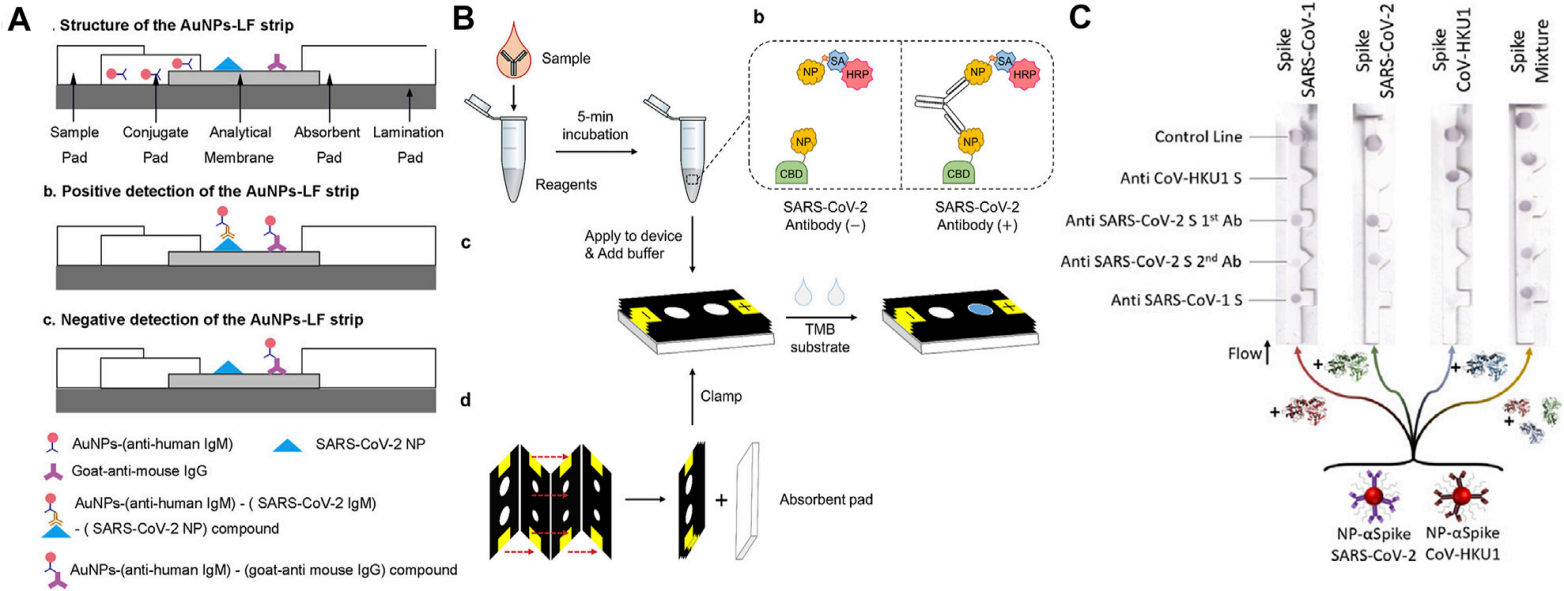
**(A)** Lateral flow paper-based antibody test. Adapted with permission from (Huang et al., 2020), under ACS Author Choice License; **(B)** antibody test using a μPAD. Adapted with permission from (Kim et al., 2021), copyright American Chemical Society 2021; **(C)** lateral flow paper-based antigen test. Adapted with permission from (Hristov et al., 2021) copyright American Chemical Society 2021.

Representative paper-based antibody and antigen tests are illustrated in Figure 3 and a summation table is given below (Table I). Generally, paper-based immunoassay tests completely satisfied the ASSURED criteria established by the World Health Organization (WHO). They have merits including low production cost, portability, ease of use, and long shelf life so that these tests are suitable for use in developing and low resource countries. However, when it comes to immunoassay tests, sensitivity and specificity have always been a concern. Other limitations also include the outcome of the tests are heavily dependent on the quality and preparation of the antibodies (Hu et al., 2017), it is difficult to obtain accurate quantitative information from immunoassay tests (Sachdeva et al., 2021a) and perform complex multiplexed assays due to a lack of fluidic control (Jia et al., 2020).

**TABLE 1:** Existing paper-based immunoassay tests.

Study	Receptor	Detection technique	Total time (excluding sample preparation)	R (min)eadout	LOD	Sensitivity/specificity	Total clinical sample size
Li et al. (2020)	Antibody	LFIA	15	Colorimetric-AuNP	did not report	88.6%/90.63%	525
Wen et al. (2020)	Antibody	LFIA	15	Colorimetric-AuNP	did not report	69.1%/100%	85
Huang et al. (2020)	Antibody	LFIA	15	Colorimetric-AuNP	did not report	100%/93.3	19
Zhou et al. (2021a)	Antibody	LFIA	10	Colorimetric- PEI-CISG AuNP	did not report	100%/100%	10
Zhou et al. (2021b)	Antibody	LFIA	10	Fluorescent-QBs	did not report	97.1%/100%	69
Chen et al. (2020)	Antibody	LFIA	10	Fluorescent-LNP	did not report	100%/88%	58
Kim et al. (2021)	Antibody	Cellulose-binding domain	15	Colorimetric-TMB-HRP	5 nM	96.2%/93.9%	3
Kasetsirikul et al. (2020)	Antibody	ELISA	20	Colorimetric-TMB-HRP	9.00 ng μl^−1^	N/A	N/A
Diao et al. (2021)	Antigen	LFIA	10	Fluorescent-FIC	did not report	75%/100%	251
Grant et al. (2020)	Antigen	LFIA	20	Colorimetric-Latex bead	0.65 ng/ml	N/A	N/A
Liu et al. (2021a, b)	Antigen	Chemiluminescence	15	Colorimetric-Nanozyme	0.1 ng/ml	N/A	N/A
Hristov et al. (2021)	Multiplexed antigen	30 min	30	Colorimetric-AuNP	0.1 nM	N/A	N/A
Yakoh et al. (2021)	Antibody or antigen	Electrochemical	30	Square voltammetry	0.14 nM	100%/90%	17

### Paper-Based Nucleic Acid Tests

Paper-based nucleic acids tests combine the specificity of PCR and the portability of POC tests. They can be fully automated and have adequate sensitivity to identify clinical suspicious nucleic acid samples in relatively low concertations and are one of the most sensitive of all available POC tests for infectious disease diagnosis (Niemz et al., 2011). Garneret and co-workers developed a NAAT to detect SARS-CoV-2 by coupling isothermal amplification to paper-based microfluidic techniques. The device possessed capabilities including nucleic acids extraction, on-chip amplification using RT-LAMP, and naked-eye visualization for qualitative analysis. In this work, nasopharyngeal swabs were collected from suspected COVID-19 cases. Also, binder-free glass fiber was used as both extraction membrane and reaction membrane. The membranes were inserted into a 3D-printed disc to complete the POC device, which has a production cost of 2–4$. To prepare the sample, sample lysis and elution steps were performed manually using commercial buffers. Real-time RT-LAMP was used for the amplification of the Orf1ab gene. A DNA intercalating dye (SYTO-82) was used for measuring the fluorescence emission. The test took approximately 1 h to complete and a limit-of-detection (LOD) of 1 copy/μl was estimated (Garneret et al., 2021). However, the report indicated that the detection of only the ORF1ab gene cannot ensure reliable diagnostics of SARS-CoV-2 (Suo et al., 2020). Zhu and co-workers developed a multiplex RT-LAMP amplification method that used two LAMP primer sets in an isothermal reaction to simultaneously amplify SARS-CoV-2 target sequences (ORF1ab and the N gene) to improve the test accuracy. By labeling the amplicon products with FITC and biotin, Dig and biotin, respectively, the ORF1ab and N genes of SARS-CoV-2 were simultaneously detected by NALFIA through immunoreactions and biotin/streptavidin affinity binding. The test showed a LOD of 12 copies/reaction, a total run time of 1 h with a 15 min manual DNA extraction step, and a 100% test specificity as well as sensitivity was demonstrated by collecting and analyzing clinical oropharynx swab samples obtained from 33 patients infected with SARS-CoV-2 and 96 non-SARS-CoV-2 infected patients (Zhu X. et al., 2020). Also using the RT-LAMP amplification approach, Zhang and co-workers developed a one-pot direct RT-LAMP assay combined with lateral flow technology for the detection of SARS-CoV-2. Different from the previous works, NaOH solution was used to lyse the collected nasopharyngeal swab samples so that target nucleic acids (SARS-CoV-2 N gene and ORF1ab Gene) were directly released for isothermal amplification without nucleic acid extraction step, thereby shortening the tests time to ∼40 min. NALFIA detection method through immunoreactions was also used in this work and the LOD was estimated to be 1 copy/μl (Zhang et al., 2021). Additionally, based on the NALF method, Yu and co-workers developed a paper device that is capable of simultaneous detection of three regions of the SARS-CoV-2 genome (RdRp, ORF3a, and the nucleocapsid (N)-protein gene) using RT-PCR amplification. The simultaneous detection of the three genes avoids cross-reactivity with other coronaviruses and possible false-negative results caused by mutations in the SARS-CoV-2 genome. The test allows the detection of SARS-CoV-2 in 30 min with a limit of detection (LOD) of 10 copies/test for each gene. However, a PCR instrument was necessary for the amplification of the genomic copies, thus limiting the possibility of the NALF assay to be developed into a POC test (Yu et al., 2020).

Other Isothermal amplification techniques have also been employed by the paper-based nucleic acid tests. Qian and co-workers developed a NALFIA test for the detection of SARS-CoV-2 N gene and S gene using a reverse-transcription recombinase polymerase amplification (RT-RPA) reaction. RPA is an isothermal amplification reaction optimally working between 37 and −42°C. It amplifies the target sequence using a recombinase, single-strand binding proteins, and a strand displacement polymerase (Magro et al., 2017). Compared with LAMP, RPA has the advantage of operating at a lower temperature. In this work, a forward and a FAM-labeled reverse pair of primers specific to the target sequence were designed for amplification. By combining RT-RPA with lateral flow technology, after mixing the reaction components with running buffer, the mixture is delivered to the detection zone where dual FAM-labeled and biotin-labeled products are detected on a lateral flow strip. The test takes approximately ∼45 min from sample collection to results and has a LOD of ∼0.5 copy/μl (Qian et al., 2020). Xia and co-workers introduced a POC test for simultaneous detection of SARS-CoV-2 S gene and N gene by integrating reverse transcription–enzymatic recombinase amplification (RT–ERA), a modified version of RT-RPA. In their experiment, Mg^2+^ was used as the ERA activator and the authors designed a pair of nfo forward and reward primers to amplify an amplicon within both N and S genes. Nfo-affinity probes were also designed for the detection of the N and S gene. The approach demonstrated a detection limit of 0.05 copy/μl. The relatively high assay sensitivity allows target SARS-CoV-2 gene to be mixed directly with diluted throat swab without extra sample processing nor RNA purification, thereby simplifying the sample preparation step and reducing the overall analysis time to ∼30 min (Xia and Chen, 2020). However, the amplification step for both works has to be completed off-chip. To address this, Liu and co-workers developed an integrated lateral flow RT-RPA assay for the detection of the SARS-CoV-2 N gene. During testing, the RT-RPA reaction components are introduced to the chip and then incubated. After incubation, amplification products and running buffer are mixed, then delivered to the lateral flow strips for easy qualitative results interpretations. This approach achieved a detection limit of 1 copy/μl and a total run time of ∼30 min (Liu et al., 2021b).

Wu and co-workers instead used a barcoded isothermal nucleic acid sequence-based amplification technique (NASBA) to develop a two-stage test. After performing a rapid diagnosis on-chip using the NASBA reaction in stage one, the end product was sent to a central facility for pooled sequencing for improved detection accuracy and detailed analysis. Also, by introducing a FAM labeled and biotin-labeled RNA capture oligonucleotides into the NASBA reaction, both oligonucleotides can bind to different parts of the single-stranded RNA NASBA product. For target gene detection, neutravidin-conjugated carbon nanoparticles (NA-CNPs) were added to the lateral flow assay. The aggregation of NA-CNPs at the test line resulted in a color change. To achieve multiplexed sequencing, a sample-specific barcode pair was incorporated into the amplicon during the NASBA reaction to facilitate the selection in the sample pool in stage two. The second stage was then used to reconfirm the initial diagnosis and enabled centralized data processing. This approach allowed a quick decentralized readout within 2 h and achieved a detection limit of <50 copies of viral RNA per reaction (Wu et al., 2021). Additionally, methods for achieving detection signal amplification other PCR, LAMP, or other isothermal target amplification techniques have been developed. Wang and co-workers demonstrated a lateral flow nucleic acid immunoassay for rapid detection of SARS-CoV-2. The underlying principle of the assay relies on the affinity binding between the S9.6 monoclonal antibody with DNA–RNA hybrid molecules. In this work, the hybridized DNA-RNA double strands were formed by the specific SARS-CoV-2 DNA probes and the lysed virus genome (ORF1ab, envelope protein, and the N-protein gene). Upon introducing the target samples, the fluorescent-nanoparticle-labeled S9.6 antibody was used to bind to the double-stranded DNA–RNA hybrids. Unlike other nucleic acid detection techniques that use targe amplification, the sensitivity of this assay highly depends on the length of the DNA probe and the molar ratio of the antibody to the hybrid. The absence of nucleic acid amplification means that the assay does not suffer from contamination by amplicons. Also, the overall testing duration can be shortened and the process can be simplified. The assay demonstrated a PCR comparable LOD of 0.5 copies/μL. In addition, the assay also achieved a 100% sensitivity and 99% specificity, thus making the assay potentially viable for POC use (Wang D. et al., 2020).

**FIGURE 4:**
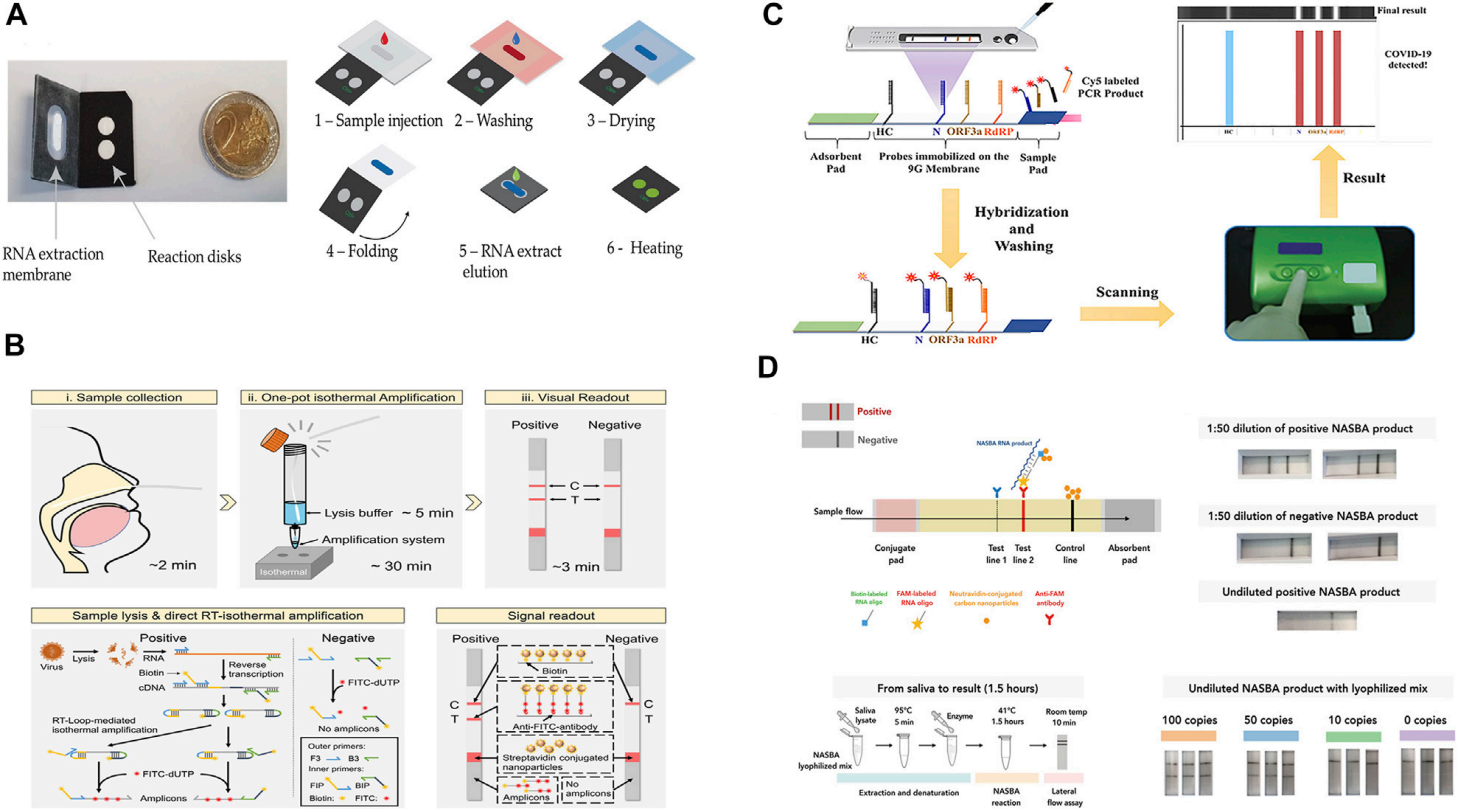
**(A)** paper-based μPAD integrated with RT-LAMP amplification. Adapted with permission from (Garneret et al., 2021) under Creative Commons license; **(B)** lateral flow combined with one-pot RT-LAMP amplification. Adapted with permission from (Zhang et al., 2021), copyright American Chemical Society 2021; **(C)** multiplex detection of SARS-CoV-2 genome. Adapted with permission from (Yu et al., 2020) copyright American Chemical Society 2020; **(D)** two-stage detection and multiplexed sequencing technique. Adapted with permission from (Wu et al., 2021) under Creative Commons license.

Representative paper-based devices are illustrated in Figure 4 and a summation table is also given below (Table 2). Although paper-based nucleic acids tests retained sensitivity and specificity of the conventional RT-PCR tests, and they represented a good solution for sample-in-answer-out testing in low-resource settings. However, like conventional PCR tests, paper-based nucleic acids tests also involve multiple physical and/or chemical steps, and existing works have not yet developed a fully integrated POC system, as one or more steps have to be completed off-chip. In this case, trained personnel are still required for administering these tests to avoid human mishandling. Therefore, designing paper-based nucleic acids tests that are fully integrated and usable by untrained users is the current goal and an unavoidable challenge that many POC tests face.

**TABLE 2:** Existing paper-based nucleic acid tests.

Study	Target gene	Amplification method	Total time (excluding sample preparation)	Extraction required	LOD	Sensitivity/specificity	Total clinical sample size
Garnt et al. (2020)	ORF1ab	RT-LAMP	1 h	Off-chip	1 copy/μl	100%/100%	21
Zhu et al. (2020a, b)	ORF1ab, N	RT-LAMP	45 min	Off-chip	12 copies/reaction	100%/100%	129
Zhang et al. (2021)	ORF1ab, N	One pot RT-LAMP	40 min (from sample collection to results)	No extraction	2 copy/μl	100%/100%	18
Yu et al. (2020)	RdRp, N, ORF3a	RT-PCR	30 min	Off-chip	10 copies/reaction	100%/99%	162
Qian et al. (2020)	N, S	RT-RPA	45 min (from sample collection to results)	No extraction	0.5 copy/μl	86.7%/100%	51
Xia and Chen (2020)	N, S	One-pot RT-ERA	30 min	Off-chip	0.05 copy/μl	N/A	N/A
Liu et al. (2021a, b)	N	On-chip RT-RPA	30 min	Off-chip	1 copy/μl	97%/100%	54
Wu et al. (2021)	S	NASBA reaction	1–2 h	Off-chip	30 copy/μl	N/A	N/A
Wang et al. (2020a, b)	ORF1ab, N	No Amplification	40 min	No	0.5 copy/µl	100%/99%	734

### Paper-Based CRISPR/Cas Tests

CRISPR/Cas systems have been gaining tremendous attention in biotechnology since the modified CRISPR/Cas9 system was applied for gene editing in mammalian genomes (Cong et al., 2013). Recently, CRISPR-Cas systems, including CRISPR-Cas12 and CRISPR-Cas13, exhibit robust collateral activity against single-stranded DNA (ssDNA) and RNA targets, respectively. When combined with a FRET-based reporter, a fluorophore connected to a quencher via a short oligonucleotide sequence, the presence of the target can be thus confirmed. Such collateral activity provides the basis for highly specific, sensitive approaches for nucleic acid detection (Ali et al., 2020a).

Additionally, by exploiting the simplicity of isothermal amplification methods, such as LAMP and RPA, the detection of a few copies of the target nucleic acid can be readily achieved. In this regard, CRISPR-based diagnostic methods have been utilized in conjunction with paper-based platforms to achieve efficient POC testing of SARS-CoV-2 genes.

Broughton and co-workers developed a Cas12a-based DNA endonuclease Targeted CRISPR Trans Reporter (DETECTR) assay with a lateral flow platform for SARS-CoV-2 detection. In this assay, Cas12 gRNAs were designed to target the N and E genes of SARS-CoV-2. Upon performing simultaneous reverse transcription and isothermal amplification using loop-mediated amplification (RT-LAMP) for viral RNA extracted from patient nasopharyngeal or oropharyngeal swab samples and control RNA targets, the Cas12a trans-cleavage activity allowed the detection of the target amplicons was achieved by cleaving the FAM-biotin reporter molecules at the test line of the lateral flow strip. Using a fluorescence-based readout, qualitative detection with a LOD of up to 10 copies/µL was achieved with an operating time of 45 min. The assay was validated by testing 82 clinical respiratory swab samples collected from confirmed COVID-19 patients and others. After comparing with standard RT-PCR tests, 95% sensitivity and 100% specificity were achieved (Broughton et al., 2020). Nguyen and co-workers developed a modified Cas12a-based assay by engineering guide RNA extensions to affect Cas12a trans-cleavage activity. The method was referred to as ENHANCE (enhanced analysis of nucleic acids with crRNA extensions) by the authors and was used for the detection of the SARS-CoV-2 N gene. The authors discovered that the extensions of guide RNA on the 3′ or 5′ end would increase the trans-cleavage activity, of which, the 3′ end with 7-mer extensions showed the highest activity. The authors contributed this improvement to the conformational changes of the LbCas12a effector induced by the engineered guide RNA. By combining with isothermal amplification of SARS-CoV-2 RNA using RT-LAMP, the modified Cas12a assay was incorporated in a lateral flow assay to detect SARS-CoV-2 viral. Similar to the previous work, the detection of the target amplicons was achieved by cleaving the FITC-ssDNA-Biotin reporter. The assay demonstrated a LOD of 3–300 RNA copies. However, no clinical samples were used for the assay validation (Nguyen et al., 2020). Ali and co-workers also developed a Cas12-based SARS-CoV-2 lateral flow detection method (iSCAN) incorporating RT-LAMP. Different from the DETECTR method, by replacing the Cas12a effector with Cas12b, the author achieved one-pot detection of SARS-CoV-2 viral, thereby further improving the assay accessibility. However, the author discovered simultaneous mixing of various components in one pot leads to a substantial reduction in detection performance and sensitivity. After optimizing the one-pot process flow by employing special tubes that allow sequential mixing of reaction components, a LOD of 100 copies per reaction was found. Also, a comparable detection sensitivity 86% and specificity was obtained by evaluating clinical samples from 21 SARS-CoV-2-positive patients and three healthy individuals (Ali et al., 2020a). Tsou and co-workers also developed a Cas12a-based SARS-CoV-2 detection method. Instead of RT-LAMP, the authors incorporated RT-RPA as the isothermal amplification technique, and the guide RNA was designed to target the S, M, and N genes of the virus. Similarly, the detection of target amplicons was achieved by cleaving the single-stranded DNA-FAM-quencher reporter at the lateral flow strip. The authors discovered that the guide RNA targeting M gene of the SARS-CoV-2 showed the highest efficiency and detection sensitivity with a LOD of 0.1 copy/μl. In addition, when cell lysate and supernatant of SARS-CoV-2 samples were directly added to the RT-RPA reaction without the RNA extraction step, a LOD of 10 copies/μl was found. The method was validated using 10 clinical nasopharyngeal swab samples of COVID-19 patients and 12 healthy individuals and an overall 100% sensitivity and 100% specificity were achieved. However, the validation experiments were performed in-tube instead of a lateral flow strip (Tsou et al., 2021). Moreover, to further improve the testing efficiency and facilitate the loss in the signal caused by viral mutations, Ooi and co-workers developed a Cas12-based method to detect wild-type and mutated SARS-CoV-2 viral. The authors discovered that the engineered effector enAsCas12a was more robust toward both the operating temperature and mismatch tolerance. Additionally, the use of modified guides improves reaction kinetics. Particularly, hybrid DNA-RNA guides would give higher collateral activities compared to regular gRNAs, thus enhancing the on-target signal while suppressing off-target background to negligible levels. The enhanced sensitivity and specificity allowed the assay to be completed without an RNA purification step. The authors first showed that the assay still worked when a S254F mutation was present in the viral template. The assay was then validated by testing nasopharyngeal swab samples obtained from 21 confirmed COVID-19 patients and 21 healthy individuals without a RNA purification step. A sensitivity of 80%, a specificity of 100%, and a LOD of 40 copies/μl (2 copies/μl for purified RNA samples) were determined with a total operating time of 30 min (Ooi et al., 2021). To resolve the current single gene detection limitation of the CRISPR-based lateral flow assay, Yin and co-workers developed a lab-on-paper platform for multiplex gene diagnosis of SARS-CoV-2. This method also combined RT-RPA and CRISPR-Cas12a detection. Paper-based CRISPR detection chambers and a 3D-printed RPA reactor were initially isolated through a normally closed sucrose valve. The detection reagents were pre-loaded in the detection chambers. After RPA amplification at a pre-set time, the sucrose valve automatically opened and amplicons migrated to the CRISPR-based detection chambers, reacting with the pre-loaded detection reagents with specific guide RNAs. The platform was capable of simultaneously detecting the N gene and S gene of the SARS-CoV-2 virus as well as a reference control in a single clinical sample with a total turnaround time of 1 h. The method was validated using RNA samples extracted from 21 nasopharyngeal swab clinical samples. A LOD of 100 copies per test was determined and 100% sensitivity and specificity were reported (Yin et al., 2021).

In addition to Cas12, Cas13a was discovered to possess the target RNA triggered trans-cleavage activity (Li et al., 2019b). Patchsung and co-workers used the specific high-sensitivity enzymatic reporter unlocking (SHERLOCK) technique for rapid detection of SARS-CoV-2. Similar to Cas12-based detection, SHERLOCK detection also relies on RT–RPA to amplify target gene segments, followed by CRISPR–Cas-mediated detection of the amplified genes. The authors designed a total of four RPA-primer pairs and the corresponding guide RNAs targeting S, N, and Orf1ab genes of SARS-CoV-2. Overall, the SHERLOCK detection system achieved 100% specificity and 88% sensitivity using SARS-CoV-2 RNA extracted from nasopharyngeal and throat swabs of infected patients with a LOD of 40 RNA copies per reaction. In addition, the technique was amenable to multiplexed detection in a single lateral-flow strip by incorporating an internal control for ribonuclease contamination (Patchsung et al., 2020). Additionally, based on the Cas 13 detection method, Li and co-workers developed a lateral flow test for targeting N genes of SARS-CoV-2. By incorporating reverse-transcription and recombinase-aided amplification (RT-RAA) with conventional colloidal gold test strip approach, this assay achieved a relatively high LOD of 1 copy/μl with a naked eye readout. However, the authors did not elaborate on the cause for this sensitivity enhancement. Additionally, to reduce the false-positive results commonly associated with the colloidal gold test strips, the authors adjusted the assay detection mode so that the cleaved FAM-biotinylated reporter molecules could only be captured at the control line but not at the test line. In this case, the authors considered the disappearance of the test band as positive, thus minimizing subjective readouts and improving the test accuracy. Validated with blind tests of 649 clinical samples, this assay achieved a test sensitivity of 90.67% and a specificity of 99.21% (Li et al., 2021). Moreover, Fozouni and co-workers developed another Cas13a-based lateral flow strip for the detection of SARS-CoV-2 viral. To make this assay applicable for POC testing, the authors did not include a nucleic acid amplification step. Instead, a combined guide RNA design targeting E and N genes was proposed to improve sensitivity and specificity. In addition, the authors used a mobile phone camera as a portable plate reader to eliminate the need for an external fluorescence plate reader. Nevertheless, the exclusion of the amplification step limited the LOD of the assay to 200 copies/μl (Fozouni et al., 2021). Azmi and co-workers on the other hand developed a Cas13a-based lateral-flow assay for the detection of SARS-CoV-2 viral in saliva. The authors used a combined method of chemical treatment and heat inactivation to release target RNA from saliva samples. RT-RPA and SHERLOCK assay was subsequently performed for the detection of the S gene. The method also incorporated a smartphone for semi-quantitative fluorescence signal on-site readout. This assay also achieved excellent sensitivity and specificity. However, a LOD of 100 copies/μl could potentially impact its utility (Azmi et al., 2021).

Cas12 and Cas13 based effectors are more frequently used in nucleic acids detection as Cas9 lacks trans-collateral activity. However, a few Cas9-based approaches have also been developed. Xiong and co-workers reported a CRISPR/Cas9-mediated lateral flow assay (CASLFA) combined with RT-RPA for multiplex detection of SARS-CoV-2 viral genes. In this case, various guide RNAs were designed for target genes recognition. Also, a scaffold sequence that contained a binding site for recruiting AuNP-DNA probes was incorporated in the guide RNA. After the biotinylated amplicons are specifically recognized by Cas9/gRNA complex, the AuNP-DNA probes will bind to the guide RNA via nucleic acid hybridization. The visible accumulation of AuNP-DNA probes at the lateral flow strip was used for naked-eye detection. Their design of the lateral flow strip included dual test lines for multiplex detection of the SARS-CoV-2 E gene and Orf1ab gene. Validated using nasopharyngeal swab clinical samples, a 97% assay sensitivity and a 100% specificity were achieved with a LOD of 4 copies/μl (Xiong et al., 2021). Additionally, previous works have shown that the Cas9 effector from the Gram-negative bacterium Francisella novicida (FnCas9) can be reprogrammed to target a specific RNA substrate (Price et al., 2015). Based on this, Azhar and co-workers developed another lateral flow test that utilized a direct FnCas9 based enzymatic readout for detecting SARS-CoV-2 viral gene sequences. Similar to an affinity-based method of detection, FnCas9 has a high specificity for mismatches. It can improve the assay’s specificity while allowing a single-mismatch sensitivity. The same RT-RPA amplification and fluorescence detection methods were used for signal transduction and readout. As a result, for a total of 46 clinical samples, a 100% assay sensitivity and a 97% specificity were achieved with a LOD of 400 copies per reaction (Azhar et al., 2021). In another work, Kumar and co-workers also adapted the FnCas9-based method for the detection of SARS-CoV-2 S gene mutation N501Y using a lateral flow strip. Because Cas9 was highly sensitive to changes in its target sequence, a small mutation in the target nucleic acid led to the protein binding less strongly, thus allowing signal transduction. The results from clinical sample tests were also promising. A sensitivity of 87% and specificity of 97% were obtained with a LOD of 400 copies per reaction (Kumar et al., 2021). Marsic and co-workers also developed a Cas9-based detection technique, named Vigilant. The technique used a fusion of catalytically inactive Cas9 (dCas9) endonuclease and VirD2 relaxase to form Cas9-VirD2 fusions that have been demonstrated to be able to efficiently bind to DNA templates and cleave DNA targets (Ali et al., 2020b). In their work, VirD2-dCas9 specifically bound the target sequence via dCas9 and covalently bound to a FAM-tagged ssDNA reporter via VirD2. A guide RNA was designed to target the SARS-CoV-2 N gene. The complementarity of the guide RNA brought the VirD2-dCas9-ssDNA-FAM to the N gene sequence. Same as the others, off-chip RT-RPA coupled with fluorescent readout on a lateral flow strip was used for nucleic acid detection. Validated using clinical samples, the Vigilant technique also achieved excellent sensitivity and specificity of 96.4 and 100% with a LOD of 2.5 copies/μl, respectively (Marsic et al., 2021).

**FIGURE 5:**
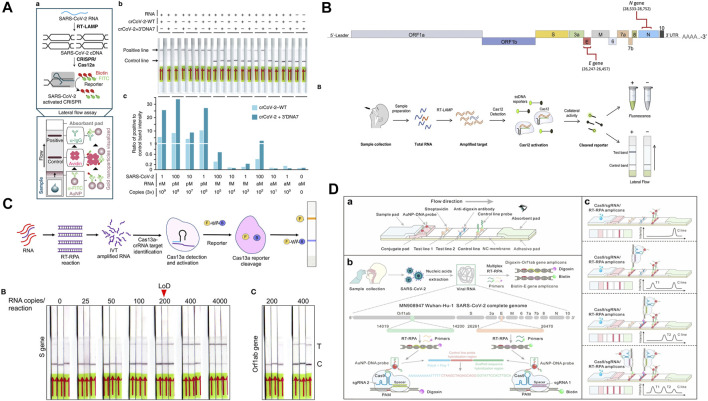
**(A)** Cas12-based ENHANCE method with RT-LAMP amplification. Adapted with permission from (Nguyen et al., 2020) under Creative Commons license; **(B)** CRISPR–Cas12a-based iSCAN assay. Adapted with permission from (Ali et al., 2020a) under Creative Commons license; **(C)** Cas13a-based lateral-flow assay. Adapted with permission from (Azmi et al., 2021) under Creative Commons license; **(D)** CRISPR/Cas9-mediated lateral flow assay. Adapted with permission from (Xiong et al., 2021) copyright Wiley-VCH 2021.

Representative paper-based CRISPR/Cas tests are illustrated in Figure 5 and a comparison table is given below (Table 3). As an emerging biosensing technology, the current CRISPR/Cas systems have successfully demonstrated the potential to develop a highly sensitive, easy-to-use, and cost-effective paper-based detection platform. Also, it is easily adaptive to a variety of diagnostic tools such as LAMP and RPA to enhance sensitivity to a few copies of the targeted nucleic acid at a single temperature in a short time. Thus, CRISPR/Cas biosensing systems are well-suited for developing paper-based POCT devices. However, it is still a rather recent approach, the practicality of this approach remains a challenge as some of the Cas proteins are still only available in laboratories. Also, the long-time storage of the reagents, which is essential to paper-based POCT, needs further study.

**TABLE 3:** Existing paper-based CRISPR/Cas tests.

Study	Target gene	Cas protein type/Amplification method	Total time (excluding sample preparation)	Extraction required	LOD	Sensitivity/specificity	Total clinical sample size
Broughton et al. (2020)	N, E	Cas12a/RT-LAMP	45 min	Off-chip	10 copies/µl	95%/100%	82
Nguyen et al. (2020)	N	LbCas12a/RT-LAMP	40–60 min	Off-chip	3–300 copies	N/A	N/A
Ali et al. (2020b)	E, N	Cas12b/RT-LAMP	1–2 h	Off-chip	10 copies/reaction	86%/100%	24
Tsou et al. (2021)	S, M, N	Cas12a/RT-RPA	1–2 h	Off-chip/or no extraction	0.1 copy/µl w extraction 10 copies/µl w/o extraction	100%/100%	22
Ooi et al. (2021)	S	enAsCas12a/RT-LAMP	30 min	Off-chip/or no extraction	2 copy/µl w extraction 40 copies/µL w/o extraction	80%/100%	42
Yin et al. (2021)	Multiplex N, S	Cas12a/on-chip RT-RPA	1 h	Off-chip	100 copies/reaction	100%/100%	21
Patchsung et al. (2020)	S, N Orf1ab	Cas13a/RT-RPA	1–2 h	Off-chip	40 copies/reaction	88%/100%	380
Li et al. (2021)	N	Cas13a/RT-RAA	1–2 h	Off-chip	1 copy/µl	90.67%/99.21%	649
Fozouni et al. (2021)	E, N	Cas13a/no amplification	40 min	Off-chip	200 copies/µl	did not report	5
Azmi et al. (2021)	S	Cas13a/RT-RPA	30 min	chemical treatment and heat inactivation	100 copies/µl	95.7%/100%	76 (saliva)
Xiong et al. (2021)	E, Orf1ab	Cas9/RT-RPA	45 min	Off-chip	4 copies/µl	97.14%/100%	64
Azhar et al. (2021)	N	FnCas9/RT-RPA	1 h	Off-chip	400 copies/reaction	100%/97%	46
Kumar et al. (2021)	S-N501Y	FnCas9/RT-RPA	1 h	Off-chip	400 copies/reaction	87%/97%	59
Marsic et al. (2021)	N	dCas9/RT-RPA	1 h	Off-chip	2.5 copies/µl	96.4%/100%	30

### Other Paper-Based Tests

Besides the most commonly used methods listed above, other paper-based tests also have been developed for the detection of SARS-CoV-2 (Table 4). Despite being unconventional, these methods may prove to be useful for testing under some specific conditions. De Silva and co-workers used paper spray mass spectrometry to detect the SARS-CoV-2 virus through analyzing lipid-related metabolomics. In this work, Teslin^®^ synthetic paper, which composed of micro-porous polyolefin-silica matrix was used as a substrate for depositing sample containing solution droplet. Compared with commonly used cellulose paper, previous work by the authors has indicated that Teslin^®^ paper allowed the samples to have a larger active signal, resulting in a higher amount of ion formation, and with less interference of molecules from the substrate (De Silva et al., 2019). By comparing with standard RT-PCR tests, the mass spectrometry method achieved a 93.3% sensitivity with an analyzing time of 1 min. Although this method is not suitable as a POCT for virus detection since it requires the use of a complex instrument and lacks further clinical validation. However, the results are particularly meaningful for providing valuable insights into the immune response of the novel coronavirus (De Silva et al., 2020).

**TABLE 4:** Other paper-based tests.

Study	Method	Amplification required	Total time (excluding sample preparation)	E (min) xtraction required	LOD	Sensitivity/specificity	Total clinical sample size
De Silva et al. (2020)	paper spray mass spectrometry	No	1	Off-chip	did not report	93.3%/NA	30
Alafeef et al. (2020)	Graphene-based electrochemical	No	5	Off-chip	7 copies/µL	100%/100%	48

Finally, in recent years there have been many reports of using 2D nanomaterials for ultrasensitive diagnosis of diseases (Zhu et al., 2015). Due to its excellent intrinsic electrical and mechanical properties (Hao et al., 2018; Wang C. et al., 2020), graphene has emerged as the most promising functional material (Luo et al., 2021). To data, graphene-based biosensors have been reported to detect a wide range of biomolecules including nucleic acids (Wang et al., 2019a), proteins (Tang et al., 2018; Hao Z. et al., 2019), and small molecules (Wang et al., 2019b; Li et al., 2019c). Based on this, Alafeef and co-workers developed a paper-based electrochemical platform to enable the rapid detection of the SARS-CoV-2 viral gene. In this work, a graphene suspension was coated on filter paper. It played an important role in the sensing response as its high carrier mobility made it highly sensitive to the interaction and absorption of the charged target at its surface. The authors designed a specific oligonucleotide sequence that was mounted on the surface of gold nanoparticles to hybridize with its complementary viral RNA. In the presence of SARS-CoV-2 RNA, the specific RNA−DNA hybridization led to the change in charge and electron mobility on the graphene surface, which brought the change in sensor output voltage. The authors also developed a hand-held reader that potentially enabled on-site measurements of the sensor’s voltage output. The platform achieved a LOD of 7 copies/μl while reducing the analysis time to 5 min because of the amplification-free detection process. The validity of the sensor platform was demonstrated with a 100% sensitivity and specificity using clinical samples gathered from COVID-19 positive patients and healthy individuals (Alafeef et al., 2020). The above methods all demonstrated feasibility in rapid, sensitive, low-cost SARS-CoV-2 detection and the potential for applying them for POC use. However, the study of these methods is still preliminary and more results in particular results for cross-interference and virus mutation detections are still needed.

## Conclusion and Future Research Prospects

It has been almost 2 years since the first reported case of COVID-19. Unfortunately, because of the highly transmissive nature of the virus and the frequent occurrence of new variant mutations, there is still no treatments available for the virus. Therefore, mass detection, timely diagnosis, and physical intervention methods such as social distancing remain as one of the effective methods to stop the virus from transmitting. However, mass detection and rapid diagnosis are difficult to achieve even in developed countries since the conventional methods for detecting viruses are extremely resource-consuming. Therefore, the development of rapid, robust, highly sensitive, and specific POC diagnostic tests is still in need. Based on such needs, this review summarized the recent developments of paper-based detection tools for the rapid diagnosis of SARS-CoV-2. Currently, the paper-based detection tools can be grouped into three major categories, nucleic acid tests, immunoassay tests, and CRISPR tests. These tests have the common advantages of being cost-effective, user-friendly, and time-efficient, thus holding a great potential to complement the conventional PCR methods. On other hand, the nucleic acids tests offer higher sensitivity and specificity, but they often are complex. CRISPR tests potentially represent the next-generation diagnostics methods. However, the method itself is still in the developmental stage, and how to integrate with paper-based platforms such as immobilizing CRISPR/Cas molecules onto paper substrate remains an outstanding issue. Finally, immunoassay tests are the simplest of them all, which is ideal for fast decision-making but lacks both sensitivity and specificity. Although novel research on paper-based tests has been making great progress to achieve reliable POC diagnostics, their applications in COVID-19 diagnosis are still hindered. In the article, we attempt to enlist and discuss some future research directions that apply to paper-based SARS-CoV-2 diagnostic tools in the hope to bring the technologies to practical use.

One of the research directions is reagent storage. In an ideal setting, reagents are stably stored within the paper devices and can be used at moment’s notice. However, most biochemical reagents can regrade and lose their functionality within weeks or even days. Also, storage strategies for reagents can vary substantially due to different degrees of reagent sensitivity to various environmental parameters such as storage temperature (Krauss et al., 2017). Although effective dry storage of specific enzymes and antibodies has been demonstrated successfully in porous materials (Wentland et al., 2021), research on the dry storage of PCR reagents and nucleic acids used in CRISPR/Cas systems are still scared. In addition, the rehydration of the stored reagents to their original functionality is equally important but is rarely discussed.

Another important research area is sample preparation. This is particularly important for nucleic acids and CRISPR tests as they often reply on amplification to enhance the detection sensitivity. However, amplification requires complex viral RNA extraction protocols that can only be done by professionals using dedicated instruments to avoid false results. This is one of the important reasons why there hasn’t been a commercially available paper-based nucleic acids POC test yet. Althrough proof-of-concept paper tests discussed in this review have shown the ability to perform extraction-free amplification and subsequent viral gene detection, they are still in the early developmental stage and more studies on the extraction-free approaches must be done before a paper-based nucleic acids test can become practically useful.

Finally, Multiplex detection is a necessity in many medical diagnostic tests as many infectious diseases have almost identical symptoms such as SARS-COV-2 and the common cold. In addition, some diseases are caused by the co-existence of several pathogens and serotypes (Safenkova et al., 2019). Therefore, medical diagnoses will benefit much more from simultaneous and rapid detection of multiple targets as opposed to a single target. Also, autonomous multistep detection in a paper device is preferred, in particular for assays that require multiple manual processing steps such as in NAATs or CRISPR/Cas systems. Thus, it can eliminate most of the manually induced detection uncertainties and significantly reduce the complexity of the assays. Past research has demonstrated different types of paper-based valves through changing the wicking property of paper, varying geometric dimensions of the paper channels, and using manually, electrically, thermally, and electromagnetically actuated valves to adjust the connectivity of fluidic channels. These works have been thoroughly reviewed (Akyazi et al., 2018). Nevertheless, most of the valves still operate manually or can only operate on a single channel. As a result, a paper-based flow-control system that is applicable for performing fully autonomous multistep assays (e.g. NAATs) is still under development. Our group recently developed a paper-based valving technology that enabled multiplexed control of parallelized paper channels with a high degree of autonomy. We hope this technology can potentially pave the way for the development of fully automated paper-based NAATs.

Through the discussion of existing works and future research perspectives, we hope that this review identified what is needed, what has been done, and what are challenges still need to be addressed. We believe through close collaboration between academia and industries, a paper-based POC test with high sensitivity and specificity while fully satisfying the ASSURED criteria is on the horizon. Although it may not be here in time to stop the COVID-19 pandemic, it certainly will have a tremendous social and economic impact on our society for infectious diseases monitoring and prevention in the future.
